# Utility of 24-hour ambulatory blood pressure monitoring in potential living kidney donors

**DOI:** 10.1186/s40885-021-00172-4

**Published:** 2021-07-01

**Authors:** Nabeel Aslam, Sobia H. Memon, Hani Wadei, Elizabeth R. Lesser, Shehzad K. Niazi

**Affiliations:** 1 Division of Nephrology & Hypertension, Mayo Clinic Florida, 4500 San Pablo Road, Jacksonville, FL 32224 USA; 2 Department of Transplantation, Mayo Clinic Florida, Jacksonville, USA; 3 Department of Biostatistics, Health Science Research, Mayo Clinic Florida, Jacksonville, USA; 4 Department of Psychiatry, Mayo Clinic Florida, Jacksonville, USA

**Keywords:** Ambulatory blood pressure monitoring, Living donors, Hypertension

## Abstract

**Introduction:**

Hypertension (HTN) is a risk factor for cardiovascular disease; therefore, it is imperative to risk stratify potential kidney donors during evaluation. Clinic blood pressure (CBP) measurement is inaccurate in assessing presence or absence of HTN. There is paucity of data about utility of 24-h ambulatory blood pressure monitoring (ABPM) during kidney donor evaluation.

**Methods:**

24-h ABPM is performed on all kidney donors at Mayo Clinic Florida. We conducted retrospective review of 264 consecutive potential kidney donors from 1/1/2012 to 12/31/2017. Demographic, comorbid conditions, laboratory results and 24-h ABPM data were collected. Subjects were divided into two groups: Group1: Subjects with no prior history of HTN and new diagnosis of HTN using 24-h ABPM; Group 2: Subjects with no prior history of hypertension and normal BP on 24-h ABPM.

**Results:**

Baseline demographic included mean age 46.40 years, 39% males, 78.4% Caucasians, and mean BMI was 26.94. Twenty one subjects (8.0%) had prior diagnosis of HTN. Among 243 subjects without prior HTN, 62 (25.5%) were newly diagnosed with HTN using 24-h ABPM. CBP was high only in 27 out of 62 (43.6%) of newly diagnosed HTN subjects. Thirty-five subjects (14.4%) had masked HTN and 14 subjects (5.8%) had white-coat HTN. Newly diagnosed hypertensive subjects were more likely to be males as compared to Group 2 (53.2% vs 34.3% *P* = 0.008). There was a trend of more non-Caucasians subjects (30.6% vs 19.9% *P* = 0.08) and more active smokers (17.7% vs 11.6%, *P* = 0.054) in Group1 as compared to Group 2. Only 17 (27.4%) out of 62 newly diagnosed hypertensive subjects were deemed suitable for kidney donation as compared to 105 (58.0%) out of 181 normotensive subjects (*P* < 0.001).

**Conclusion:**

In our cohort, use of ABPM resulted in new diagnosis of HTN in 1 out of 4 potential kidney donors. Newly diagnosed HTN was more common in men, those with non-Caucasian race, and in active smokers. There was a significantly reduced acceptance rate for kidney donation among newly diagnosed HTN subjects. Further studies are needed to determine the value of 24-h ABPM among these high risk groups.

## Introduction

Living kidney donation is critically important in renal transplantation because of the large number of end-stage renal disease patients on the kidney transplant waiting list [1]. Potential kidney donors need to carefully weigh several risk factors before donation. This risk-benefit analysis to reach a well-considered and informed decision to proceed or not with kidney donation is dependent upon effective identification of relevant risk factors that impact post-kidney donation outcomes [2]. To do so, a comprehensive pre-kidney donation assessment is needed. However, assessment practices vary across transplant centers. Undiagnosed hypertension (HTN) is one such factor that impacts future kidney health. Additionally, HTN is an independent risk factor for cardiovascular disease [3–5]; therefore, accurately diagnosing HTN is a cornerstone in potential kidney donor evaluation.

Clinic blood pressure (CBP) measurement is the most common method of identifying hypertension; however, office based blood pressure measurement has several limitations due to measurement technique errors and inherent variability in the blood pressure [6]. It is uncertain whether BP measured in the transplant clinic accurately detects presence or absence of hypertension among kidney donors. On the other hand, blood pressure measurement done in a busy clinic practice can result in an overestimation or underestimation of the blood pressure, thus leading to over or under diagnosis of hypertension [7]. Furthermore, CBP is unable to detect the white coat and masked hypertension [8, 9]. Twenty-four hours ambulatory blood pressure monitoring (ABPM) is a technique for evaluating out of office blood pressure over the 24-h period [10]. It can reliably diagnose hypertension and identify both white coat and masked hypertension [6, 11]. ABPM recording also provides data on the diurnal changes in the BP [12]. Despite its apparent advantages, utilization of 24-h ABPM in evaluating living kidney donors has not been universally adopted by transplant centers partially due to paucity of data about the utility of ABPM during kidney donor evaluation.

In the general, diagnosis of HTN is missed in about a quarter of the population and the prevalence of white coat HTN and masked HTN further complicate the matters [6]. This phenomenon is equally likely to affect potential kidney donors. If a kidney donor has a new diagnosis of HTN it may change the risk-benefit analysis and thus impact the decision of the donor to proceed with the donation or the transplant center’s decision to accept the patient for donation. However, this question has not been systematically studied so far. Our center has relied on 24-h ABPM in diagnosing HTN in all living kidney donors since 2003. We sought to retrospectively examine our center’s experience with 24-h ABPM in diagnosing HTN compared to CBP measurement among potential kidney donors as well as to evaluate the impact of new diagnosis of hypertension on acceptance for kidney donation.

## Materials & methods

The study was approved by institutional review board. Mayo Clinic Florida has integrated electronic health records (EHR) with access to kidney donor candidates’ work up. Subjects were identified from query of billing records for kidney donor evaluation between 01/01/2012 to 12/31/2017 at Mayo Clinic Florida where all kidney donor candidates undergo 24-h ABPM as part of routine pre-kidney donation evaluation. We retrospectively collected data from EHR on demographics, comorbidities, history of tobacco use, medications, office BP as well as data from 24-h ABPM, and laboratory results. Office BP was measured by clinical staff using auscultatory method with a wall aneroid sphygmomanometer (Welch Allyn, Skaneateles Falls, New York, USA) at the time of kidney donor evaluation visit at the transplant clinic. A cut-off value of systolic BP ≥140 mm (Hg) or diastolic BP ≥90 mm (Hg) was used to define elevated BP based on office BP measurement.

Spacelabs ABPM monitor and software (Spacelabs healthcare, Snoqualmie, WA, USA) was used for ABPM. BP was measured automatically and stored every 10 min during awake hours and every 20 min during nocturnal hours during 24-h ABPM. Subjects were determined to have new diagnosis of hypertension if they had no prior history of hypertension and/or not on antihypertensive medications at the time of donor evaluation, and if one of the following occurred during the 24-h ABPM: mean daytime systolic blood pressure > 135 mmHg, mean daytime diastolic blood pressure > 85 mmHg, mean nighttime systolic blood pressure > 120 mmHg, mean nighttime diastolic blood pressure > 75, overall 24-h mean systolic blood pressure > 130, overall 24-h mean diastolic blood pressure > 80.

White-coat hypertension was defined as clinic systolic BP ≥ 140 mm (Hg) or diastolic BP ≥ 90 mm (Hg) and ABPM systolic and diastolic B*P* values outlined above not meeting the criteria of new diagnosis of hypertension. Masked hypertension was defined as clinic systolic BP ≤ 140 mm (Hg) and clinic diastolic BP ≤ 90 mm (Hg) and ABPM exceeding the systolic or diastolic BP outlined above for the diagnosis of hypertension. Subjects were divided into two groups: Group 1: Subjects with no prior history of hypertension and new diagnosis of hypertension using 24-h ABPM during donor evaluation; Group 2: Subjects with no prior history of hypertension and normal BP on 24-h ABPM.

## Statistical analysis

Data were summarized with the mean (SD), for numeric variables and number (percent) for categorical variables. Chi-square was used to look for significant differences and relationships between categorical variables amongst groups. The Kruskal Wallis test was used to assess significant differences for continuous variables. All statistical tests were two sided and *p*-values less than 0.05 were considered statistically significant. All analysis was performed in the R Statistical Software (version 3.6.2; R Foundation for Statistical Computing, Vienna, Austria).

## Results

**Fig. 1:**
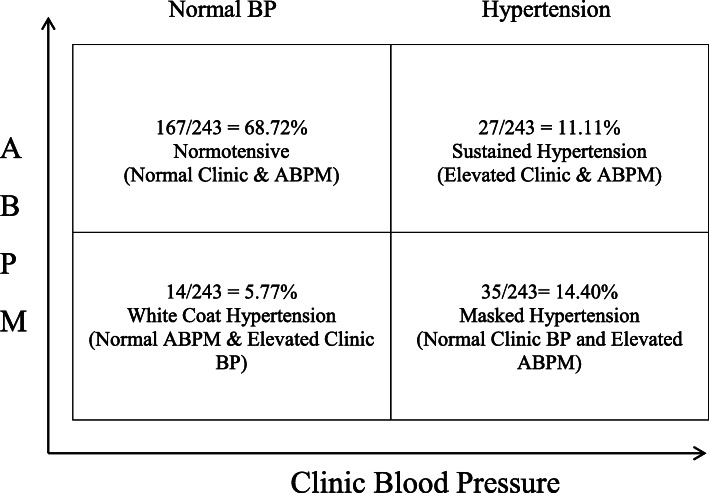
Blood Pressure Phenotype based upon ABPM

Total of 264 subjects underwent kidney donor evaluation between 01/01/2012 to 12/31/2017 at Mayo Clinic Florida. Baseline demographics included mean age 46.40 ± 13.13 years, 39.0% males, 78.4% Caucasian, mean BMI 26.93 ± 6.72, and history of tobacco use 39.8% (Table 1). Twenty one subjects out of 264 (8.0%) had prior history of hypertension at the time of donor evaluation. These subjects were excluded from any further analysis. The remaining 243 subjects out of 264 (92.0%) had no history of hypertension at the time of donor work up. 24-h ABPM identified 62 (25.5%) out of 243 subjects with new diagnosis of hypertension based upon criteria outlined above and 181(74.5%) out of 243 subjects were normotensive. Office BP was elevated in 27 (43.65%) out of 62 of newly diagnosed hypertensive subjects by ABPM whereas office BP was normal in 35 (56.4%) out of 62 of newly diagnosed hypertensive subjects. White coat hypertension was present in 14 (5.8%) and masked hypertension was diagnosed in 35 (14.4%) out of 243 subjects (Fig. 1). Among the cohort of 62 newly diagnosed hypertension, 13(21.0%) had diastolic hypertension alone, 21 (33.9%) had systolic hypertension alone, and 28(45.2%) had both systolic and diastolic hypertension using 24-h ABPM.

**Table 1:** Baseline Characteristics Stratified by Newly Diagnosed Hypertension, Normotensive Groups, and Overall group Using 24-h Ambulatory BP Monitoring

	New Hypertension (*N* = 62)	Normotensive (*N* = 181)	Overall (*N* = 264)	*P* value
Age, years	46.65 (12.43)	45.01 (13.25)	46.40(13.13)	0.37
Sex, Male	33 (53.2%)	62 (34.3%)	103 (39.0%)	0.008
Race, Non-Caucasian	19 (30.6%)	36 (19.9%)	57 (21.6%)	0.081
Caucasian	43 (69.4%)	145 (80.1%)	207 (78.4%)	
Ethnicity, Not Hispanic nor Latino	54 (87.1%)	162 (89.5%)	237 (89.8%)	0.78
Body Mass Index	26.24 (6.11)	26.78 (7.08)	26.93 (6.72)	0.38
Prior history of Hypertension	0 (0.0%)	0 (0.0%)	21 (8.0%)	N/A
Diabetes	1 (1.6%)	0 (0.0%)	1 (0.4%)	N/A
Dyslipidemia	4 (6.5%)	17 (9.4%)	22 (8.3%)	0.48
Cardiovascular Disease	8 (12.9%)	25 (13.8%)	34(12.9%)	0.86
Pulmonary Diseases	8 (12.9%)	17 (9.4%)	28(10.6%)	0.43
Gastrointestinal (GI)	8 (12.9%)	16 (8.8%)	30(11.4%)	0.36
Genitourinary Disorder (GU)	5 (8.1%)	12 (6.6%)	20(7.6%)	0.70
Neurologic Disorders	10 (16.1%)	23 (12.7%)	37(14.0%)	0.50
Adjustment Disorders	3 (4.8%)	17 (9.4%)	22(8.3%)	0.26
Anxiety disorders	2 (3.2%)	15 (8.3%)	18(6.8%)	0.18
Mood Disorders	3 (4.8%)	6 (3.3%)	9(3.4%)	0.58
Smoking History				0.054
Active	11 (17.7%)	21 (11.6%)	34(12.9%)	
Former	9 (14.5%)	53 (29.3%)	71 (26.9%)	
Never	42 (67.7%)	107 (59.1%)	159(60.2%)	
Serum Creatinine, mg/dL	0.86 (0.19)	0.84 (0.18)	0.85 (0.18)	0.72
Accepted for Organ Donation	17 (27.4%)	105 (58.0%)	129 (48.9%)	< 0.001

Normal dipping pattern was defined as ≥10% drop in nocturnal systolic and diastolic BP as compared to awake systolic and diastolic BP readings. Non-dipping was defined as < 10% decrease in nocturnal systolic and diastolic BP as compared to awake systolic and diastolic BP. There was no statistically difference between the hypertensive and normotensive groups for abnormal dipping pattern (42.6% vs 36.1%; *p* = 0.36).

The comparison of demographics, comorbidities, and serum creatinine between Group 1(*n* = 62; new HTN diagnosed based on ABPM without prior diagnosis of HTN) and Group 2 (*n* = 181; no prior HTN and normotensive based on ABPM) are summarized in Table 1. Newly diagnosed hypertensive subjects were more likely to be males as compared to Group 2 (53.2% vs 34.3% *P* = 0.008). Trends observed consisted of more non-Caucasians race subjects (30.6% vs 19.9% *P* = 0.081) and more active smokers (17.7% vs 11.6%, *P* = 0.054) in Group1 as compared to Group 2. There was no other statistical difference between the groups (Table 1).

There was a clinical and statistically significant difference in the percentage of subjects who were accepted for kidney donation among the newly diagnosed hypertensive and normotensive groups. Only 17 (27.4%) out of 62 newly diagnosed hypertensive subjects were deemed suitable for kidney donation as compared to 105 (58.0%) out of 181 normotensive subjects (*P* < 0.001) Table 1. 7 out of 14 (50%) subjects with white-coat hypertension were accepted for kidney donation. Among the masked hypertension subjects, 7 out of 35 (20%) were deemed eligible for kidney donation.

The reasons for denial were listed in EHR as medical conditions or psychosocial issues. Among the newly diagnosed hypertension group, 45 out of 62 subjects were deemed ineligible for kidney donation (37 subjects for medical reasons alone, 5 for both medical and psychosocial reasons, and 3 undocumented reasons). Among the normotensive group, 76 out of 181 subjects were deemed ineligible for kidney donation (52 subjects for medical reasons, 3 for both medical and psychosocial reasons, 14 for psychosocial reasons, and 7 undocumented reasons). There was a statistically significant difference between hypertensive and normotensive groups for denial due to medical reasons (67.7% vs 30.4%; *p* < 0.001), whereas there was no statistically significant difference for denial due to psychosocial issues (8.1% vs 9.4%; *p* = 0.75). Furthermore, there was no statistically significant difference between the groups for other comorbid conditions (Table 1), suggesting that the new diagnosis of hypertension may have been a significant factor in determining eligibility for kidney donation.

## Discussion

In this single center retrospective study that used large number of potential living kidney donors, we were able to demonstrate that utilizing 24-h ABPM accurately classified hypertension in about 20% of the cases. We also identified that male gender and potentially non-Caucasian race and smoking history were risk factors of undiagnosed hypertension in living kidney donors. Use of 24-h ABPM resulted in new diagnosis of hypertension in approximately 1 out of 4 potential kidney donors.

Potential kidney donors weigh risk factors before donation, but assessment varies across centers [13]. It is critical to identify risk factors that impact post-kidney donation outcomes to help potential donors make an informed decision. Undiagnosed hypertension is one such factor that impacts cardiovascular and renal outcomes. Office BP measurement is not always reliable. In our cohort, office blood pressure alone at the time of kidney donor work up missed more than half of the cases of undiagnosed hypertension and would have potentially placed them at risk of future adverse cardiovascular and renal outcomes. While the actual number of living donor evaluation performed in the US during the same study time frame is currently unknown, it is imperative to consider the benefit of relying on 24-h ABPM in this population as 24-h ABPM can help not only diagnose hypertension but also uncovers white coat and masked hypertension [6] . Despite its benefits, 24-h ABPM is underutilized in the evaluation of potential living donors and has not been adopted by the majority of transplant centers around the country. One probable reason for underutilizing 24-h ABPM is the lack of information regarding its benefits in this population. The current study adds to the growing literature that supports the use of 24-h ABPM in this important at-risk population. Another potential reason for lack of relying on ABPM is the lack of insurance coverage as Medicare covers ABPM only in the case of white coat hypertension. Hopefully, with the increasing evidence of its benefit, insurance companies will extend coverage to kidney donors.

In the current study we diagnosed white coat hypertension in 14 (5.8%) of 243 potential kidney donors. While this number is seemingly small, this finding has important implications. By ruling out hypertension, we were able to proceed with the kidney donation in 7 out of 14 subjects (50% similar to normotensive group 58%) with all the subsequent benefits on the respective recipients. It also avoided the unnecessary use of anti-hypertensive medications, potential stress of carrying the hypertension diagnosis, and overburdening the medical health system. One important observation is that masked hypertension was 2.5 times as high in our cohort than white coat hypertension. This contradicts with previous studies [14, 15] that examined 24-h ABPM in potential living kidney donors which demonstrated higher rates of white coat hypertension than masked HTN. It is notable to mention that in these 2 studies almost 90% of the studied population was Caucasian while non-Caucasian race constituted 21.6% of our cohort. Although the definition of HTN utilized could have also affected the number of patients with white coat hypertension, we defined HTN in accordance with published guidelines and our definition of HTN was similar to the one utilized by Textor and colleagues [14]. Irrespective of the etiology of higher rates of masked HTN in our cohort, our study indicates that a small but significant number of potential living donors do have masked HTN and therefore should be refrained from kidney donation unless their HTN is better controlled. Future studies should assess the proportion of masked HTN in non-Caucasian living kidney donors compared to their Caucasian counterparts.

Our study has important clinical implications. We acknowledge that 24-h ABPM might not be universally available in all transplant centers. In the current study, we were able to identify male gender, non-Caucasian race, and active smoking history as the population at risk in which a 24-h ABPM will be most beneficial. Even in situations where a 24-h ABPM is not available, we believe that in this at risk group relying solely on single clinic BP is not sufficient and other methods of measuring BP such as home BP monitoring or automated office BP measurement, which averages out multiple office BP readings, should be utilized. Indeed, previous studies have demonstrated that home BP and in office automated BP measurements do correlate with 24-h ABPM readings [16, 17].

In the general population, there is limited data about the use of 24-h ABPM to screen for hypertension in the absence of abnormal office blood pressure readings. Our cohort, which represents a relatively healthy population that is volunteering to donate an organ, provides a unique opportunity to estimate the magnitude of undiagnosed hypertension in the community.

The new diagnosis of hypertension may result in careful recalculation of risk benefit ratio for donor candidacy by both the transplant team and the potential donors. In our cohort, only 27.4% of subjects who had newly diagnosed hypertension were ultimately accepted as suitable candidates for organ donation as compared to 58.0% of normotensive subjects. Our study is limited by the lack of available data on whether new diagnosis of hypertension resulted in the unwillingness of donor candidates to further pursue kidney donation or the transplant team deciding not to accept as donors based upon the risk benefit ratio or the combination of the two factors. Over the last few years, there is an increasing acceptability of potential donors with controlled hypertension as suitable candidates [18]. Whether this willingness on part of the transplant team translates into new diagnosis of hypertension is not known. Further studies are needed to determine the value of 24-h ABPM in optimizing the cardiovascular risk stratification of potential donors.

The strengths of our study include large sample size, availability of data on office and ABPM, and detailed evaluation of co-morbid conditions. Our study is limited by its retrospective nature and the lack of specific reasons for donor denial. We are encouraged; however, that we included a large number of subjects that included larger proportion of non-Caucasian donors compared to previously published reports. While the definition of HTN in the general population has evolved over the period of the study, we used a unified definition over the 5-year study period for sake of simplicity and to facilitate statistical analysis. Future studies in living kidney donors using the current ACC/AHA criteria for diagnosing HTN should be performed to confirm or refute our results.

## Conclusions

In conclusion, using 24-h ABPM in a large number of living kidney donors that included 21.6% of the cohort of non-Caucasian race, we were able to demonstrate that relying on clinic BP alone did misclassify HTN in 20% of cases. We were also able to demonstrate that male gender, non-Caucasian race, and smoking history are risk factors for HTN misclassification and we recommend that scrutiny in measuring BP in these groups of potential kidney donors should be undertaken. Masked HTN was more prevalent in our cohort compared to previously published reports. Future studies should be directed to determine if the prevalence of masked HTN varies by donor race.

## Data Availability

The datasets during and/or analyzed during the current study available from the corresponding author on reasonable request.
